# Short Text Messages to Encourage Adherence to Medication and Follow-up for People With Psychosis (Mobile.Net): Randomized Controlled Trial in Finland

**DOI:** 10.2196/jmir.7028

**Published:** 2017-07-12

**Authors:** Maritta Välimäki, Kati Anneli Kannisto, Tero Vahlberg, Heli Hätönen, Clive E Adams

**Affiliations:** ^1^ Department of Nursing Science Turku Finland; ^2^ Turku University Hospital Turku Finland; ^3^ Hong Kong Polytechnic University Hong Kong China (Hong Kong); ^4^ Department of Nursing Science University of Turku Turku Finland; ^5^ Satakunta Hospital District Pori Finland; ^6^ Department of Biostatistics University of Turku Turku Finland; ^7^ Institute of Mental Health Division of Psychiatry University of Nottingham Nottingham United Kingdom

**Keywords:** text messaging, psychotic disorders, randomized controlled trial, medication adherence

## Abstract

**Background:**

A text messaging service (short message service [SMS]) has the potential to target large groups of people with long-term illnesses such as serious mental disorders, who may have difficulty with treatment adherence. Robust research on the impact of mobile technology interventions for these patients remains scarce.

**Objective:**

The main objective of our study was to investigate the impact of individually tailored short text messages on the rate of psychiatric hospital readmissions, health care service use, and clinical outcomes. In addition, we analyzed treatment costs.

**Methods:**

Between September 2011 and November 2012, we randomly assigned 1139 people to a tailored text message intervention (n=569) or usual care (n=570). Participants received semiautomated text messages for up to 12 months or usual care. The primary outcome, based on routinely collected health register data, was patient readmission into a psychiatric hospital during a 12-month follow-up period. Secondary outcomes were related to other service use, coercion, medication, adverse events, satisfaction, social functioning, quality of life, and economic factors (cost analysis).

**Results:**

There was 98.24% (1119/1139) follow-up at 12 months. Tailored mobile telephone text messages did not reduce the rate of hospital admissions (242/563, 43.0% of the SMS group vs 216/556, 38.8% of the control group; relative risk 1.11; 95% CI 0.92-1.33; *P*=.28), time between hospitalizations (mean difference 7.0 days 95% CI –8.0 to 24.0; *P*=.37), time spent in a psychiatric hospital during the year (mean difference 2.0 days 95% CI –2.0 to 7.0; *P*=.35), or other service outcomes. People who received text messages were less disabled, based on Global Assessment Scale scores at the time of their readmission, than those who did not receive text messages (odds ratio 0.68; 95% CI 0.47-0.97; *P*=.04). The costs of treatment were higher for people in the SMS group than in the control group (mean €10,103 vs €9210, respectively, *P*<.001).

**Conclusions:**

High-grade routinely collected data can provide clear outcomes for pragmatic randomized trials. SMS messaging tailored with the input of each individual patient did not decrease the rate of psychiatric hospital visits after the 12 months of follow-up. Although there may have been other, more subtle effects, the results of these were not evident in outcomes of agreed importance to clinicians, policymakers, and patients and their families.

**Trial Registration:**

International Standard Randomized Controlled Trial Number (ISRCTN): 27704027; http://www.isrctn.com/ISRCTN27704027 (Archived by WebCite at http://www.webcitation.org/6rVzZrbuz).

## Introduction

Mobile technology has the potential to promote patient self-management, leading to better health behaviors, which, in turn, improve clinical outcomes among people with chronic illnesses. Short message service (SMS) text messaging on mobile telephones could address some of the chronic disease management needs. Text messaging reminders are simple, efficient options that enable direct or indirect communication between health services and patients in a time- and cost-efficient manner [1]. The majority of mHealth studies have tested basic mobile phone features, such as text messaging [2], but the effectiveness of using text messaging for supporting treatment adherence among people with serious mental disorders, such as schizophrenia, is still not clear [3].

The use of mHealth apps is expanding in various areas of health care [4], including mental health [5], and likewise, the evaluation of such apps is increasing [1]. Nevertheless, clear evidence of the value of mHealth apps in improving adherence, within of the realm of chronic disease management, is still lacking [6]. With regard to people with schizophrenia, for whom adherence is particularly an issue [7], the relevant up-to-date Cochrane review found that information and communication technology (ICT)-based prompts were not clearly beneficial: in 1 randomized controlled trial with 320 participants, the relative risk of stopping medication within 6 months was 1.11 (95% CI 0.96-1.29; moderate-quality evidence) [8]. Despite this, the number of mHealth apps promoting medication adherence has increased, including apps specifically targeted at improving mental health [9]. The potential harm that such technology can cause for people with chronic mental illnesses must also be considered [10], although very few studies have specifically addressed this issue [11]. Some patients find text messaging disturbing and discontinue reading the messages [12]. In theory, this could increase the sense of isolation among such patients, resulting in decreasing adherence [13]. The effects of SMS text messaging on mental health and health service use also remain incompletely explored. To provide precise results of the effects of text messaging on clinically relevant outcomes, high-grade, longer-term, and adequately powered studies are needed [14].

In this paper, we report the findings of a large pragmatic, multicenter, parallel-group, 12-month randomized controlled clinical trial investigating the impact of individually tailored SMS text messages on the rate of psychiatric hospital readmissions, health care service use, and clinical outcomes. We hypothesized that patients in the intervention group receiving SMS prompts would have fewer psychiatric hospital readmissions during the 12-month follow-up period, less service use, fewer coercive incidents, less medication use, fewer adverse events, higher satisfaction, higher social functioning, a better quality of life, and lower treatment costs, compared with those who did not receive SMS prompts. Our assumption behind our hypothesis was that if text message reminders lead to improved adherence to treatment—for example, to better medication intake, appointment keeping, or self-management—then we would see fewer hospital readmissions or other adverse events.

## Methods

### Design and Study Setting

The Mobile.Net study was initiated by the Academy of Finland to assess the effectiveness of SMS text messaging in encouraging medication compliance and self-care for people with serious mental illnesses. We assumed that when a patient is willing to take their medication frequently as prescribed, the positive results can be seen as a decreased need for health service use. Good patient medication adherence reduces the rate of relapse and hospital readmissions [15]. The rationale, design, and methods have previously been described [16]. The trial was undertaken at psychiatric hospitals in Finland. We approached the managers of the hospitals, sent them written material about the study, and allowed them up to 3 weeks to decide if they wanted their hospital to participate. Of 30 possible hospital organizations, 24 organizations (45 wards) decided to take part. The trial was approved by the Ethics Committee of the Hospital District of Southwest Finland (December 16, 2010; ETMK 109/180/2010).

### Participants

Participants in the trial were adults (aged 18-65 years) at the point of discharge from a psychiatric hospital ward, for whom ongoing antipsychotic medication had been advised. Each participant had a mobile phone, was able to use the Finnish language, and gave written informed consent to participate. We did not include a formal test of capacity, but rather, we relied on the judgment of experienced health care professionals in their routine assessment of patients’ understanding, retention, assimilation, and communication of information as patients were nearing the point of discharge. Formalized assessment of patient cognition is not part of routine psychiatric care in Finland. We excluded people who had a planned nonacute treatment period in a hospital ward, as well as those who were being treated in forensic psychiatric services. Participants gave written informed consent to take part in the study, complying with the Declaration of Helsinki, the World Health Organization principles of good clinical practice, and national requirements.

### Randomization and Masking

We used a central randomization service at the University of Turku (the Department of Mathematics and Statistics). The study was individually randomized, open label, stratified by hospital, with a variable (random) permuted block length of 4 patients per block, to ensure that trial groups at each hospital were balanced. The allocation was computer generated by an independent statistician outside of the study and masked to participants. The investigators who enrolled participants could not foresee assignment. In addition, the statisticians, the outcome assessors of the survey data, the data analysts, and the National Register holder—responsible for the Finnish routine data used in this study—were kept blinded to allocation. However, after randomization, due to the type of intervention, the allocation group was unmasked to participants, the research nurses at each hospital, and the health care staff on the wards. While the Data Management Committee undertook ongoing safety surveillance, investigators running preliminary analyses for the Committee were masked to data until the database was released.

The high number of hospitals and study wards included in the trial necessitated that recruitment be undertaken in 10 waves, between September 2011 and November 2012. A research assistant, completely independent of the trial team (masked for allocation), inserted the allocation numbers into sealed envelopes. Written allocations of assignment, sealed in entirely opaque individual envelopes and marked with a study identification number, were distributed to each study ward. Research nurses on each study ward sequentially assigned the sealed envelopes in a predetermined order to people who had both fulfilled the inclusion criteria and given their written informed consent during their discharge process.

### Recruitment

We used standardized, face-to-face informed consent procedures for patient recruitment during the inpatient stays, before a patient was discharged from the ward. Nurses were asked to identify potentially eligible patients from the medical records. These patients were given a short, 1-page information leaflet about the study, and then, at their time of discharge, patients were provided with more detailed written information with an invitation to participate. If willing, patients then attended appointments with a research nurse (specifically trained for this task) to discuss practical arrangements, check eligibility, and complete a baseline assessment (age, sex, marital status, vocational education, employment status, and number of psychiatric treatment periods) and study registration. Before consenting, participants were made aware that they were free to withdraw without obligation at any time and that such an action would not adversely affect any aspect of their care. We did not envisage that the intervention would interfere with routine outpatient care.

### Interventions

Patients in the experimental group received semiautomated 1-way text messages for up to 12 months from the time of recruitment. To increase acceptability of the prompts and engage users, the fundamental content of the 85 text messages was designed by both service users and health care professionals [16], and then tailored with input from each individual patient at the point of randomization. Patients in the intervention group selected compulsory text messages regarding medication (eg, “Take your medication, please,” “It is important to take your medication as prescribed.”) and treatment appointments (“Remember to book a follow-up appointment,” “Please go to your follow-up appointment.”), as well as voluntary text messages related to their free time and daily management (“Are your clothes clean and tidy?”, “Be more gentle with yourself.”). Text message examples are translations from the Finnish language. The content of the text messages is described in greater detail in Kauppi et al [17].

Messages were selected in cooperation with the research nurses on each ward and were recorded in a text message booklet. Text message selection was based on patients’ preferences: each patient was able to select the exact messages he or she wanted [17]. There was no specific or predefined schedule for the messages. However, to make sure patients did not receive an overwhelming amount of text messages, and to prevent patient habituation to the text messages [18], we limited the total amount of text messages to up to 12 messages in a month. The patients were able to choose the amount (between 2 and 12 text messages per month), time (any time, day or night) and day of the week (from Monday to Sunday) of the selected messages (see Kauppi et al [17]). The messages were not personalized in any other way, to protect patient privacy. Patient names and information related to illnesses or medication were not mentioned in the messages. The text messages were not interactive (they were 1-way messages), and therefore no response was required from patients. If a patient sent a message to the research group, we reviewed it and contacted staff members responsible for that particular patient if needed. Otherwise, the patient would receive a general response, unrelated to their treatment. Text messages were sent to the patients automatically via a specific digital text message reminder system. Table 1 provides a detailed description of the intervention [19,20].

Research nurses (n=129) who worked on the study wards had 2 days of training, which covered theoretical and practical issues in randomization and intervention management, and two 1-day training updates during the trial. Each research nurse recorded the number of patients in each ward, the eligible participants, the number of those who refused, and those who gave informed consent. Every 2 weeks, Mobile.Net researchers from the research center (in Turku, Finland) made quality control assessment phone calls to each of the 45 wards to ensure that eligible patients were assessed, baseline data collected, and ethical requirements followed. These calls also monitored whether staff had received any messages from patients or their caregivers describing discomfort or harm caused by the text messages and to identify any problems in randomization. In addition, we visited the research wards at least twice during the recruitment period: we held face-to-face meetings with the research nurses and staff members to ensure high-quality data collection, maintained interest, and an *esprit de corps*. Email support for the study ward was also available during the months of patient recruitment.

**Table 1:** Description of the short message service (SMS) text messaging intervention^a^.

Categories	Description
Brief name	Tailored, patient-led SMS intervention.
Rationale and theory	The intervention is based on self-determination theory [20], which explains human motivation. The theory assumes that developing a sense of autonomy, competence, and relatedness is critical to processes of internalization and integration, through which an individual comes to self-regulate and maintain behaviors beneficial to health and well-being. Intrinsic forms of motivation involve engaging in behaviors for their own sake (eg, for challenge and enjoyment), while extrinsic forms of motivation involve doing an activity because it is instrumental in achieving a separate consequence. Environments that promote autonomy and support confidence are likely to enhance compliance and health outcomes.
Materials	Materials (and their users) were a computer with access to the Internet at the hospital (research nurses); mobile phone with a SIM^b^ card to receive text messages (patients); a paper-format text message library, including a list of 85 text messages with the contact information of the research nurse and researchers (research nurses, patients).
Procedures	The patients selected their favorite text messages from a text message library. The research nurse input each person’s preferences of text messages into an electronic semiautomated system.
Providers	The semiautomated system was managed by nurses in psychiatric hospitals.
How?	Messages continued for 12 months or until participants no longer wanted to receive the messages. The messages did not include any personal health-related content (eg, identification codes or names, diagnoses, medication, or name of health services). Participants were advised to inform researchers or research nurses if their mobile number changed, if they felt at all uncomfortable because of the received text messages, or in case of any technical problems.
Where?	Patients received SMS messages after being discharged from psychiatric hospital.
When and how much?	The timing, frequency, and conditions under which SMS messages were to be stopped were decided by the participants. The total number of messages received, free of charge to patients, was limited to a maximum of 12 per month or 4 per week (the minimum was 2 per month related to medication).
Tailoring and modifications	Patients were able to stop or change the topic, frequency, or timing of any messages by sending an email, telephoning, text messaging, or mailing researchers or staff members.

^a^Modified from the template for intervention description and replication (TIDieR) checklist and guide, Hoffmann et al [19].

^b^SIM: subscriber identity module.

All patients allocated to standard care (control group) continued with usual care after discharge from hospital at the discretion of their psychiatric and nursing team. The health professionals were able to use any resources at their disposal to offer maximum care for patients based on the existing system in Finland, which does not automatically include regular text messages. We did not restrict the use of any technological applications for these people.

### Outcomes

#### Primary Outcome

The intervention in the experimental group involved a maximum of 12 months of SMS text messaging. We collected all of the data for the primary outcome from routine data collection by the Finnish national Care Register for Health Care (HILMO; formerly the Finnish Hospital Discharge Register) [21]. The register includes individual clinical and administrative data of all people treated in psychiatric hospitals in Finland. The completeness and accuracy of this register have been found to vary from “satisfactory” to “very good” [22]. To our knowledge, and that of those administering the register, Mobile.Net is the first trial to use these routinely collected data for outcomes for a randomized trial. The use of the register as the main outcome source was important, as the focus of the study was to illustrate the use of information on health utilization for consideration by the multiple stakeholders for whom these routine data are collected—especially health providers [23].

The primary outcome was patient readmission to a psychiatric hospital (ie, how many patients [n, %] in each study arm were readmitted to a psychiatric hospital during the 12-month follow-up). The other outcomes related to service use were (1) time to next hospitalization (how many days a patient was out of the psychiatric hospital after discharge [days]), (2) time in a psychiatric hospital during the year (total number of days admitted in a psychiatric hospital during the 12-month follow-up period), and (3) healthy time (number of days during the 12-month follow-up period when the person was not admitted in a psychiatric hospital [days]). Each participant was followed up for 12 months after discharge.

#### Secondary Outcomes

Secondary outcome data (also taken from routinely collected data in the national register) included type of admission (n, %), involuntary treatment (number of periods of care), general hospital treatment, length of involuntary psychiatric treatment (days), length of general hospital stay (days), use of private care (data not available), coercion used (yes, no), and type of coercive incidence according to the Finnish Mental Health Act (1116/1990; seclusion, limb restraint, forced injection, physical restraint), medication use (yes, no), type of medication (antipsychotic, antipsychotic and antidepressant), and adverse events (any [yes], death according the Statistics of Finland [yes]). We assessed other secondary outcomes, patient satisfaction with care or intervention (Client Satisfaction Questionnaire-8 [CSQ-8], self-rated) [24], and quality of life (Quality of Life Enjoyment and Satisfaction Questionnaire [Q-LES-Q], self-rated) [25] with a structured survey supplemented by a postal or telephone survey 12 months after randomization. Patient disability was assessed by patient functioning (Global Assessment Scale [GAS], 0-100, staff rated) [26], also taken from routinely collected data in the national register. Outcomes concerning patient engagement in the intervention (fidelity) were assessed by whether or not a patient made a “request to stop SMS” (intervention group only) or “leave the study early” (for any reason), relayed by the patient or clinical staff members and recorded by the investigators after informed consent.

We analyzed treatment-emergent adverse events, defined as any harm or adverse events occurring between randomization and when the patient completed the study, reported by anyone involved. Information was collected by emails, telephone calls, text messages (staff, patients, or relatives), and visits or face-to-face meetings with staff members and recorded using a standardized instrument originally based on the clinical research monitoring and good clinical practices network [27]. We categorized harms as unexpected or expected, at different levels (a severe adverse event or an adverse event) [28]. Severe adverse events were life-threatening (an event during which the patient was at risk of death) or fatal, required inpatient hospitalization, prolonged hospitalization, or resulted in a major disability [28]. We further categorized the adverse events into medical or psychiatric problems or substance use.

We also ascertained research nurses’ and patients’ perceptions regarding possible harms in using SMS (“In your opinion, have text messages caused any harm to you/to patients?”, yes, no). We interviewed all research nurses by telephone and interviewed patients (in the intervention group) by telephone or postal survey [29]. We collected information on patient deaths from the national health register (causes of death register) [30]. We also surveyed patients’ feedback on the intervention [29].

#### Cost Analysis

We examined the costs of the SMS intervention by calculating the unit costs of staff time used for the intervention and text message cost for each patient during the 12-month trial. We prospectively estimated staff time (45 minutes per patient and an additional 15 minutes to upload the messages into the system). We estimated mean salary and overhead costs for staff members (€3300 average monthly costs, including additional staff costs of 23.25%, about €21.21 per hour) as referred to in locally and nationally agreed-upon unit costs by the Union of Health and Social Care Professionals in Finland [31]. The costs of the text messages were assessed based on the total number of text messages sent to each patient during the 12 months (total 67,560); the cost of each text message to Finnish consumers in 2011 was approximately €0.0004 [32].

To assess the direct costs of patient care, we prospectively collected the treatment costs per day in a psychiatric hospital. The average treatment costs for nonspecialized care wards (€408 per day per patient) and specialized care wards (€692 per day per patient) were based on the nationally agreed-upon service costs by the Ministry of Social Affairs and Health in 2011 [33]. For indirect costs, the data were not available.

### Statistical Analysis

#### Power

Our primary analysis was based on a comparison of two estimates between study groups at the 12-month follow-up. We identified no comparable studies to assist our calculations. To show the difference in reducing readmissions to hospitals by at least 5 percentage points (a relative risk of 0.92) with 80% power at a 5% 2-sided significance level, we estimated that we would need a total of 1511 participants in each of the 2 arms (Stata v10; StataCorp LP). In 2009, based on HILMO, there were 8339 people with schizophrenia (*International Classification of Diseases, 10th Revision* [ICD-10] block F20-29) admitted to psychiatric hospitals who used antipsychotic medication. We hoped to be able to achieve this sample size in Mobile.Net [16]. Funding limitations compelled us to have a window of 15 months for recruitment.

#### Analysis

We carried out the analyses using the SAS System for Windows (version 9.4; SAS Institute). The primary analysis was intention-to-treat. We did not impute missing outcome data because only 2% of the data were missing for the primary outcome. For the secondary outcomes, we did not impute the data either, even though about half of the data were missing (regarding quality of life and satisfaction). The reason was that the data were collected at only 1 time point. We summarized descriptive characteristics of patients by study group, and estimated either mean (SD), median (interquartile range), or numbers and proportions as appropriate. Descriptive statistics were used to evaluate outcomes at the end point and differences between groups. The primary and secondary outcomes were involved in the calculation of relative risk (RR), odds ratio (OR), and median differences (Hodges-Lehmann estimate) or mean differences and their corresponding 95% confidence intervals. We took dependency between periods of care, measured from the same participant, into account using generalized estimating equations. Variables with yes or no answers were analyzed with a Poisson regression model, while for other dichotomous variables, binary logistic regression was used. We compared mean differences between groups using a 2-sample *t* test and median differences using a Mann-Whitney *U* test. Using data from the primary outcome alone, we investigated the effect of the SMS messaging for subgroups of people with schizophrenia-like illnesses in comparison with all other diagnostic labels. We considered 2-sided *P*<.05 to be statistically significant.

### Governance and Role of the Funding Source

The funder approved the design of the study, but had no role in the design, data collection and analyses, data interpretation, content of the manuscript, or submission for publication. A total of 4 interim reviews with the Management and Safety Committee were organized at 12, 14, 19, and 27 months after recruitment began. As expected, data were not available at those times to test the primary outcome of the hypothesis, so the Committee based their consideration of when to stop patient recruitment on analyses of recruitment speed (the number of patients recruited each day), patient allocation in each group, and refusal rates. The Management and Safety Committee oversaw the study. The corresponding author had full access to all data and was ultimately responsible for the decision to submit the manuscript for publication. TV and KAK also had access to raw data in the study.

The trial is registered with the ISRCTN registry (27704027; Multimedia Appendix 1 [34].

## Results

**Figure 1:**
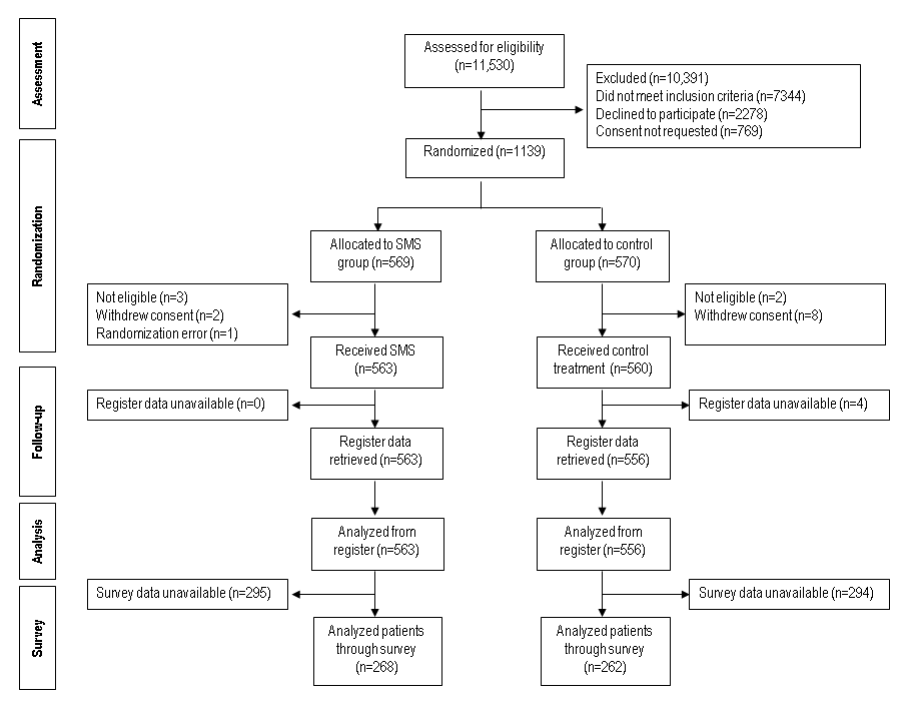
Flow diagram of study participants. SMS: short message service.

During the 15 months of recruitment (September 5, 2011 to November 30, 2012) a total of 11,530 patients were admitted onto the 45 wards. Of 4186 potentially eligible patients, 3417 (81.63%) were invited to participate. The other 769 people became ineligible because either they moved to another ward before discharge or, upon further investigation, it was unclear if they had the capacity to consent and participate or refuse participation in the study (Figure 1). Key characteristics differing between eligible and noneligible participants were that eligible participants were younger and a larger proportion were women (*P*<.001) [35]. One ward closed during the study period, but recruited participants continued in the trial in another ward without disruption.

In total, 1139 patients were randomly assigned to either the experimental group with text messages (n=569) or the control group (n=570). We excluded 16 patients from the data because of a randomization error or because they withdrew consent or were not eligible. Of the remaining 1123 people, the follow-up was conducted with 563 (563/569, 98.9%) participants in the intervention group and 560 (560/570, 98.2%) allocated to standard care. After further quality checks of register data, we excluded 4 people because of coding errors that could not be corrected; therefore, data for these patients were not available. This left 1119 (1119/1139, 98.24%) people for analysis. For the survey-based outcomes (satisfaction, quality of life), however, the data were available for 268 patients in the intervention group (268/563, 47.6%) and 262 in the usual care group (262/560, 46.8%) (Figure 1).

Table 2 shows the main characteristics of patients at baseline and at the 12-month follow-up in the intervention (n=569) and control groups (n=570). An equal number of female and male patients participated (mean age 38 years). About half were single and most of the participants had a low level of education. Participants’ mean age at the time of the first contact with mental health services was 27 years. The largest diagnosis group was F20-29 (421/1050, 40.10%, schizophrenia, schizotypal and delusional disorders, ICD-10; Table 2).

**Table 2:** Patient characteristics.

Characteristic		SMS^a^ (n=569)	Usual care (n=570)
**Age in years, mean (SD)**			
	At trial entry	38.5 (13); n=569	38.0 (12); n=569
	At first contact with services	27.4 (12); n=562	26.9 (11); n=560
Female sex, n (%)		301/569 (52.9)	278/570 (48.8)
**Marital status, n (%)**			
	Single	277/565 (49.0)	309/569 (54.3)
	Married	154/565 (27.3)	151/569 (26.5)
	Divorced	120/565 (21.2)	99/569 (17.4)
	Widowed	14/565 (2.5)	10/569 (1.8)
**Vocational education, n (%)**			
	None	177/561 (31.6)	172/564 (30.5)
	Vocational training courses	89/561 (15.9)	79/564 (14.0)
	Primary vocational skill certificate	161/561 (28.7)	162/564 (28.7)
	Secondary vocational skill certificate	80/561 (14.3)	90/564 (16.0)
	University degree	54/561 (9.6)	61/564 (10.8)
**Employment status, n (%)**			
	Employed	108/560 (19.3)	99/561 (17.6)
	Retired	269/560 (48.0)	277/561 (49.4)
	Self-employed	15/560 (2.7)	12/561 (2.1)
	Student	60/560 (10.7)	68/561 (12.1)
	Job seeker	108/560 (19.3)	105/561 (18.7)
**Number of psychiatric treatment periods, n (%)**			
	1	9/455 (2.0)	12/387 (3.1)
	≥2 treatment periods or more	446/455 (98.0)	375/387 (97.0)
**Diagnosis (ICD-10^b^block), n (%)**			
	Organic, including symptomatic, mental disorders (F00-F09)	1/535 (0.2)	2/520 (0.4)
	Mental and behavioral disorders due to psychoactive substance use (F10-F19)	31/535 (5.8)	29/520 (5.6)
	Schizophrenia, schizotypal and delusional disorders (F20-F29)	212/535 (39.6)	209/520 (40.2)
	Mood [affective] disorders (F30-F39)	161/535 (30.1)	163/520 (31.3)
	Neurotic, stress-related and somatoform disorders (F40-F49)	48/535 (9.0)	40/520 (7.7)
	Behavioral syndromes associated with physiological disturbances and physical factors (F50-F59)	1/535 (0.2)	4/520 (0.8)
	Disorders of adult personality and behavior (F60-F69)	71/535 (13.3)	63/520 (12.1)
	Mental retardation (F70-F79)	4/535 (0.7)	6/520 (1.2)
	Disorders of psychological development (F80-F89)	3/535 (0.6)	2/520 (0.4)
	Behavioral and emotional disorders with onset usually occurring in childhood and adolescence (F90-F98)	1/535 (0.2)	2/250 (0.4)

^a^SMS: short message service text message intervention group.

^b^ICD-10: *International Classification of Diseases, 10th Revision*.

**Table 3:** Primary and secondary outcome analysis.

Outcomes				SMS^a^ (n=563)	Usual care (n=556)	Coefficient (95% CI)	*P* value
**Primary outcome, n (%)**							
	Readmission to psychiatric hospital			242/563 (43.0)	216/556 (38.8)	RR^b^ 1.11 (0.92 to 1.33)	.28
**Secondary outcomes**							
	Time to next hospitalization (days), median (IQR^c^)			110.0 (44.0-14.0); n=242	104.5 (39.5-197.0); n=216	MdD^d^ 7.0 (–8.0 to 24.0)	.37
	Time in psychiatric hospital during the year (days), median (IQR)			30.0 (12.0-67.0); n=242	24.0 (10.0-67.0); n=216	MdD 2.0 (–2.0 to 7.0)	.35
	Healthy time (days), median (IQR)			330.5 (285.0-350.0); n=242	338.0 (293.5=352); n=216	MdD –4.0 (–10.0 to 1.0)	.08
	**Type of admission (number of periods of care), n (%)**						
		M1^e^ referral		78/442 (17.6)	77/396 (19.4)	OR^f^ 0.89 (0.57 to 1.38)	.59
		Mental examination		0/442 (0)	1/396 (0.3)		
		Determination of treatment		0/442 (0)	0/396 (0)		
		Other		364/442 (82.4)	318/396 (80.3)	OR 1.14 (0.74 to 1.77)	.54
	Involuntary treatment (number of periods of care), n (%)			91/439 (20.7)	95/390 (24.4)	RR 0.85 (0.61 to 1.18)	.33
	Use of private care			N/A^g^	N/A		
	Length of general hospital treatment (days), median (IQR)			0.0 (0.0-0.0); n=563	0.0 (0.0-0.0); n=556	MdD 0.0 (0.0 to 0.0)	.13
	Length of involuntary treatment (days), median (IQR)			18.0 (5.0-61.0); n=76	23.0 (5.0-79.0); n=67	MdD –2.0 (–13.0 to 2.0)	.35
	**Coercion, n (%)**						
		Coercive incidence (number of periods of care)		27/442 (6.1)	25/396 (6.3)	RR 0.97 (0.52 to 1.78)	.92
		**Type of coercive incidence (number of periods of care)**					
			Seclusion	20/443 (4.5)	25/396 (6.3)	RR 1.12 (0.57 to 2.20)	.75
			Limb restraint	12/443 (2.7)	9/396 (2.3)	RR 1.19 (0.37 to 3.80)	.77
			Forced injection	2/443 (0.5)	1/396 (0.3)	RR 1.79 (0.16 to 19.46)	.63
			Physical restraint	6/443 (1.4)	4/396 (1.0)	RR 1.34 (0.38 to 4.70)	.65
	**Medication, by type, n (%)**						
		Antipsychotic		125/241 (51.9)	117/216 (54.2)	RR 0.96 (0.74 to 1.23)	.74
		Antipsychotic + antidepressant		131/241 (54.4)	109/216 (50.5)	RR 1.08 (0.84 to 1.39)	.57
	**Adverse event, n (%)**						
		Any^h^ (yes)		6/563 (1.1)	3/556 (0.5)	RR 1.97 (0.49 to 7.90)	.34
		Death (yes)		11/563 (2.0)	16/556 (2.9)	RR 0.68 (0.31 to 1.46)	.32
	**Satisfaction with care/intervention/trial**						
		Satisfied with care (CSQ-8^i^ [24]), mean (SD)		22.4 (5.0); n=268	23.1 (5.0); n=262	MD^j^ –0.69 (–1.50 to 0.18)	.12
		Request to stop SMS^k^, n (%)		24/563 (4.3)	N/A^l^		
		Left the study early^m^, n (%)		295/563 (52.4)	294/556 (52.9)	RR 0.99 (0.84 to 1.16)	.91
	**Social functioning, n (%)**						
		**Disability^n^ (GAS^o^ [26])**				COR^p^ 0.68 (0.47 to 0.97)	.04
			46-100	106/442 (24.0)	71/394 (18.0)		
			31-45	223/442 (50.5)	189/394 (48.0)		
			1-30	113/442 (25.6)	134/394 (34.0)		
		Quality of life (Q-LES-Q^q^ [25] end point/change), mean (SD)		0.59 (0.18); n=268	0.59 (0.17); n=262	MD 0.00 (–0.03 to 0.03)	0.80
	**Economic factors, mean/median (IQR)**						
		Direct treatment costs^r^ (€) (all)		10,103/28 (26-9410); n=563	9210/0 (0-6936); n=556	MdD 26.8 (26 to 27)	<.001
		Direct treatment costs (€) (readmitted patients)		23,469/13,080 (5331-29,314); n=242	23,707/10,200 (4284-31,774); n=216	MdD 845 (794 to 3132)	0.25
		Indirect cost (€)		N/A	N/A		

^a^SMS: short message service text message intervention group.

^b^RR: relative risk (Poisson regression).

^c^IQR: interquartile range.

^d^MdD: median difference (Hodges-Lehmann estimate, Mann-Whitney *U* test).

^e^M1 referral: referral for observation.

^f^OR: odds ratio (logistic regression).

^g^N/A: not available.

^h^Monitored by study investigators.

^i^CSQ-8: Client Satisfaction Questionnaire-8.

^j^MD: mean difference (2-sample *t* test).

^k^Contact with the research team.

^l^N/A: not applicable.

^m^Did not return survey questionnaire.

^n^Data from health register.

^o^GAS: Global Assessment Scale.

^p^COR: cumulative odds ratio (<1 indicates less disability in the SMS group).

^q^Q-LES-Q: Quality of Life Enjoyment and Satisfaction Questionnaire

^r^Treatment cost per patient (€1=US $ 1.13, September 2015).

Table 3 provides estimates of treatment effects on primary and secondary outcomes. Contrary to the preliminary assumption, tailored mobile telephone text messages did not reduce patients’ use of health services in a psychiatric hospital (ie, readmission rate, the primary outcome). In total, there were 838 readmissions in the data. On the participant level, 40.93% (458/1119) of participants were readmitted to a psychiatric hospital during the follow-up period (242/563, 43.0% of the SMS group vs 216/556, 38.8% of the control group, RR 1.11, 95% CI 0.92-1.33). Receiving the text messages did not have any clear effect on time spent in hospital, time between hospitalizations, or number of days in the year that the person was thought to be well (healthy days).

No differences in other secondary outcomes were detected. Regarding the survey base outcome, patients’ satisfaction with care (mean 22.4 for the intervention group vs 23.1 for the control, *P*=.12) or quality of life (mean 0.59 vs 0.59, *P*=.80) did not differ statistically between the groups, while the response rates dropped to 47.6% (268/563) and 46.8% (262/560) in the intervention and control groups, respectively. People who received text messages were, however, less disabled (based on GAS scores) at the time of their hospital readmission than were those who did not receive text messages (OR 0.68, 95% CI 0.47–0.97).

We analyzed patient engagement with the SMS text message intervention. In total, 35 of 1123 participants contacted researchers before the end of the 12-month follow-up to report any changes or wanting to leave the study early [35]. Patients’ behavior regarding their text message selection was measured during the study [17]. In all, 5.9% (33/563) of the participants wanted to change the topic, receiving time, or frequency of the SMS text messages. The reasons for the changes included erroneously entering a message into the semiautomated system, being dissatisfied with the topic or timing of the messages, or simply wanting to stop or choose a new message [17]. Altogether, 95.2% (536/563) of the participants in the intervention group continued the SMS text message intervention throughout the entire 12 months, and 4.8% (27/563) dropped out of the intervention [35]: 3 participants dropped out before the intervention started, and 24 dropped out during the intervention period. Reasons for dropping out included (8/27) disliking the 1-way nature of the messages, finding the messages to be irritating, or no longer finding the messages to be beneficial (a more detailed description is found in Kannisto et al [35] and Kauppi et al [17]).

**Table 4:** Adverse events reported during the trial.

Adverse events		SMS^a^ (n=563)	Usual care (n=560)
**Adverse events, n (%)**			
	Any	6 (1.0)	3 (0.5)
	Mild	1 (0.2)	1 (0.2)
	Moderate^b^	1 (0.2)	0
	Severe^b^	4 (0.7)	2 (0.4)
Expected, severe		0	0
**Unexpected, severe**			
	Life-threatening or fatal^b^	3 (0.5)	2 (0.4)
	Requiring or prolonging hospitalization	0	0
	Major disability^b,c^	1 (0.2)	0
Expected, less severe		0	0
**Unexpected, less severe**			
	Medical	1 (0.2)	0
	Psychiatric (paranoid thoughts)	2 (0.4)	1 (0.2)
	Substance use	0	0

^a^SMS: short message service text message intervention group.

^b^Not consequence of study.

^c^Physical injury, not linked to study.

We also calculated the incremental cost of patient treatment per rehospitalization period. The cost of treatment was higher for people in the SMS group than the cost for the control group (mean €10,103 vs €9210, *P*<.001). When we calculated the cost of treatment for those who were readmitted to hospital, the statistically significant difference disappeared (mean €23,469 vs €23,707, *P*=.25).

We conducted a subgroup analysis for the primary outcome to compare people with schizophrenia-like illnesses versus those with other diagnoses. The analysis showed that people allocated to the SMS group who did not have schizophrenia-like illnesses had more psychiatric hospital days (median 26.5 vs 18.5, *P*=.047) and fewer healthy days (median 336 vs 345, *P*=.02) than patients in the control group during the 12 months. For the subgroup made up only of people with schizophrenia-like illnesses, we identified no clear differences.

During the 12-month follow-up, 9 adverse events were reported to the research staff (0.80% of 1119 participants). A total of 3 patients had paranoid experiences focused on the mobile phones (2 mild events reported by nurses and 1 moderate event reported by the patient by telephone or text messages to the researcher staff, involving 2 from the SMS group and 1 from the control group). One person in the intervention group had serious physical problems because of a stroke, and another participant wanted to stop the study. A total of 5 deaths were reported to the research staff (0.45%, 3 in the SMS group, with no discernible link to the text messages) (Table 4).

At the end of the study, we surveyed people allocated to the SMS group (n=403) to ask how they reacted to the intervention. A total of 51 participants (51/403, 12.7%) said that the text message intervention caused them harm. For example, some felt the text messages came too early in the day, others were irritated because of the interruption in work or leisure time, and some complained that the memory of the mobile phone filled up too quickly. More women than men perceived harm (35/223, 15.7% vs 16/178, 9.0%, *P*=.05). About three-quarters of the participants (274/383, 72%) were satisfied with the text message intervention. Two-thirds (247/385, 64%) were willing to receive text message intervention in the future (for more details, see Kannisto and colleagues [29]). In all, 13.8% (8/58) of the research nurses surveyed thought that the messaging caused some harm to patients by, for example, exacerbating paranoid thoughts. More female than male nurses thought that the text messages caused harm to patients (8/41, 19.5% vs 0/17, 0%, *P*=.05).

## Discussion

### Principal Findings

This was a pragmatic, multicenter, parallel-group randomized controlled trial, of, for this subspecialty, a large group of people with serious mental illnesses [16]. Our main assumption was that the SMS intervention, aimed at creating a higher awareness of health and well-being of individual patients, could change behavior patterns and subsequent service use. However, at the 12-month follow-up on patient service use, we did not find any advantages for the patients who used text messaging over the patients who received standard care. A Cochrane review [8] also did not find evidence that prompts increased patient adherence to treatment. The review found that ICT-based prompts were not clearly beneficial. There was weak evidence within this review suggesting small positive effects of SMS use on measures of the mental state, insight, and quality of life of patients, but the clinical significance of these data remains unclear. There have been few similar studies for this client group and few overlapping with Mobile.Net, and relevant trials were small with measure outcomes far upstream in the care flow. Findings from Mobile.Net serve to increase uncertainty whether SMS messaging, in this context, has any discernible effects.

### Interpretation of the SMS Results

Contrary to the hopes of many that mHealth will solve problems such as limited reach and access, high costs, and low effectiveness in the delivery of health care and other conditions, this does not seem to be the case.

We had hoped that text message reminders would lead to improved adherence to treatment, which, in turn, would be seen as better medication intake, appointment keeping, or self-management and, downstream, result in fewer hospital readmissions or other adverse events. We could have overestimated the power of a simple, 1-way, tailored, minimally intrusive, technical approach to change behavior. Of course, such an intervention is not enough to solve complex problems for people with serious mental health problems. but it might have helped engagement in care. Perhaps a more personalized 2-way communication and personal support would work. We had no way of tracking patient behavior between sending a text message and the health utilization outcomes in routine data. More research into each patient’s adherence level by, for example, detailed analysis of their health behavior, meetings with staff members, or medication intake, would deepen understanding of the impact of text message reminders on patient adherence and health utilization. Notwithstanding this, the simple SMS messaging we used did not work for important downstream outcomes of importance to health care providers.

We can also speculate about other reasons why the SMS intervention did not have a discernible effect on the chosen available outcomes. Although, in our study, patient engagement was high and people selected large numbers of text messages, we cannot be sure how many participants actually received, noted, or followed the content of the text messages. People may also have closed their telephone accounts or changed their prepaid systems during the intervention [36], or simply stopped reading the text messages at some point during the trial period [13]. These reasons seem improbable, though, and if they applied in this study, there would have most likely been some suggestion of one or both possibilities in our closing survey.

The one minor finding favoring the SMS intervention suggested that people allocated to the text messaging group had better global functioning scores (the Global Assessment of Functioning, collected as routine data) when readmitted to psychiatric hospital care. This is consistent with low-grade evidence from the Cochrane review, where there seemed to be some indication that ICT-based prompts had small positive effects on patient insight [8]. Our result could also have been a chance finding, as people in need of psychiatric treatment may not eagerly seek help out of fear of stigmatization [37], because they believe that treatment would be unhelpful, or as a result of poor insight [38]. Another study [39] found that around 75% (95% CI 72%-76%) of patients discontinued their medication within 18 months of follow-up. However, this should be studied in more detail, as even significant findings can also happen by chance.

In our study, the difference between groups in the lengths of involuntary treatment periods and patients’ stays in a psychiatric hospital were not statistically significant. Of all patients admitted to psychiatric hospitals in Finland in 2013 (N=26,561), less than two-thirds (29%) were involuntary admitted [40]. At the same time, those admitted to Finnish psychiatric hospitals had a 2- to 3-fold higher mortality rate than the general population in Sweden and Denmark [41]. Further, the total number of care days in specialized psychiatric care came to 1,262,253 (38,384 treatment periods), and the average number of care days for patients with schizophrenia spectrum psychosis (F20-29) was 60 days in 2013. If the length of the stay for each hospitalized patient could be decreased by 1 day with any effective and less-expensive intervention, it would mean substantial savings all the way up to the Finnish annual health budget, and it would also affect the quality of life for individual patients. Therefore, in the realm of health services, small changes can have big impacts.

### Strengths and Limitations

This study has several strengths. By using health registers as the main source of outcomes, the trial caused minimal extra burden for people with the illnesses and their nursing staff. The use of standardized outcome data reduced the possibility of response or dropout bias, and if there were such errors, it is unlikely that they would differ between the randomization groups. The participation rate of hospitals was high (24/30, 80%), and the participation rate for eligible patients was acceptable (around one-third), given the study population; that is, patients with serious mental illness being discharged from hospital. Randomization was successful, based on the evidence that patients in the intervention group and the usual care group were comparable on various indicators. The intervention itself was patient centered, in both its development (involving both end users and providers) and its execution (patients could choose the messages they were to receive). The duration of the follow-up period was 12 months, and the proportion of missing data gathered in the national health register was exceptionally small. When we added in our own questionnaire, outside of routine data, however, compliance with data acquisition did decrease, although it was still reasonably high (530/1123, 47.20%), considering the target population of the study. Mobile.Net allowed for the acquisition of high-grade data involving a detailed data monitoring system, which gathered information about different hospital wards using case notes, monitoring sheets, and frequent telephone calls to staff members throughout Finland, as well as the use of routine data from HILMO [21].

On the other hand, this study has its limitations. First, we did not achieve the target sample size: we estimated that we would need a total of 1511 participants in each of the 2 arms, and in this sense our study was powered as planned. With the achieved sample size, an 8-percentage-point difference in readmissions to hospitals between groups could be detected with 80% power at a 5% significance level. In this regard, our sample size is still adequate for drawing reliable conclusions.

Second, even though the majority of participants did not report negative effects, 12.7% (51/403) of participants in the SMS group and 13.8% (8/58) of the nurses associated with the intervention expressed beliefs that the text messages caused some harm to some participants. More women than men perceived possible harm in receiving SMS reminders [29]. We may assume here that some participants’ negative perceptions may indicate a lack of tailoring of specific messages, or raise questions on the type of interaction or suitability of the intervention as experienced by the participants. For example, 2-way communication could have given valuable opportunities to the participants to communicate with their treatment team to receive support when needed. Despite some negative perceptions of text messages, about three-quarters of the participants were satisfied with the SMS text message intervention [29]. Whether our finding can be generalized to a wider population needs to be examined further.

Third, regarding the patient-focused questionnaires and outcomes, such as patient satisfaction with care and quality of life, the low follow-up rate for patient self-assessed instruments (530/1123, 47.20%), although in keeping with trials among this patient group [42], may bias our study results for these particular outcomes. Those participants who did not participate in follow-up surveys may have been dissatisfied and therefore did not answer the survey. There was also a significant statistical difference between the participants who did not answer the follow-up survey and those who did fill out the questionnaires.

Fourth, the condition of the patient may have had some effect on their attrition. For example, those with the lowest capacity level and, therefore, perhaps with the greatest need for supervision of medication intake, could have been self-excluding. However, we could not verify this, as we had no formal test to assess capacity for patients who participated or did not participate in the study. On the other hand, we recruited patients to the study at the time of their discharge process, and therefore we may assume that the patients’ capacity to be discharged from the psychiatric hospital would have already been assessed by the psychiatrists responsible for the patients’ treatment.

Fifth, we hypothesized that, if patient medication adherence is better after the SMS intervention, the number of readmissions might be lower in the intervention group. We measured patient adherence by service utilization rather than by counting pills [43], or examining computerized prescription refill records [44] or blood tests, which may limit the sensitivity of the measurement. We decided to measure care utilization outcomes because our focus was on these outcomes, which can offer usable information for health service providers and societies for their decision making [45] and practicality in evaluating new technology [46], and, importantly, can help to avoid using methods that are invasive and place a burden on patients. Despite potential problems in using care utilization as an indication of the impact of the intervention, these outcomes are used as indicators in the data from “real practice,” for policy initiatives [46], and as proxy outcomes for economics. There is also a lack of studies addressing questions related to delivery of mental health services in randomized trials [47]. Tracking patient medication adherence would still have been highly informative about why simple SMS text messages did not work on downstream outcomes but, we suggest, greatly undermined the finding by promoting attrition. Objective investigation would have increased our certainty regarding adherence to medication, but this would have greatly threatened the pragmatic nature of the trial. A lack of close monitoring of the messages received by individual patients could therefore have hindered the fidelity of the study, and a lack of tracking of a similar type of technology in a control group should be interpreted as a study limitation. The specificity of the study may also be questioned in regard to its design, intervention, or outcomes to ascertain which of the multiple factors affected patient behavior. Each aspect of this complex intervention could then have been subsequently open to further evaluation. Perhaps the tailoring was indeed helpful, but our timing offset any positive effect. Each factor could now be tested in further trials.

Sixth, a simple semiautomated SMS text message is not at all a simple intervention. It involves the act of messaging, the nature of the message, and timing. Our study population was perhaps one of the most challenging, and also vulnerable for new interventions. At the same time, evidence for effective interventions to guide practitioners is scant, despite high levels of need and costs of care for this group of patients [48]. The selection of our study population can be defended due to its high impact on health services and costs globally [49]. We are also aware of the challenges of this particular patient group, who have capacity problems [50], are less experienced in using health technology [51], and are less motivated to engage in their treatment [52], which may further limit the use of the new technological intervention. However, it is important to test all options for care in this group, especially like the one in this study, which could be implemented without evaluation. Had such an intervention been successful, even a small improvement may have had wide repercussions in the utilization of health services.

Our results still indicate that it is feasible to provide an intervention, scalable for a wide group of people, that can be delivered by a simple technological solution. This type of intervention may make it possible to expand care provision without being limited by specific service hours, staff motivation, or availability of professionally trained health care staff. On the other hand, because this intervention did not result in absolute benefits of the service use, and yet did include possible negative effects of mHealth on patients with serious mental illness, we are compelled to draw attention to the following point. Many hopes and promises are being attached to mHealth, in that it could solve problems in health care services, such as human resources, limited access, high costs, and the difficulty of satisfying individual needs. There is also a great deal of belief and investment in mHealth technology in several service systems, conditions, and environments. Perhaps semiautomated prompting text messages, particularly, are not the answer for patients with serious mental health problems; a better alternative could be to allocate resources to investigating alternative mHealth solutions or other interventions to solve care utilization problems. We need to know the limitations of technology use for various user groups, and when to invest in exploring other strategies. This study also demonstrates how using routinely collected health data sets proved to be possible within randomized trials for important outcomes of clinical and public health value. This may well be possible elsewhere, and not only confined to countries known for culturally acceptable comprehensive data collection.

### Lessons Learned

Here are 7 things we would do differently in the future. First, the patient inclusion criteria should be even more inclusive to ensure a very large sample size. Second, negative perceptions related to SMS interventions should be more thoroughly investigated, and such potential problems should be considered in future intervention development for different mobile apps. Third, the intervention would have to be modified in the future to include 2-way communication and collaboration with staff (although currently still problematic), and an increase of supportive, visual, or voice-based elements could offer more interesting, albeit expensive, additions to the simple approach we tested. Fourth, other patient-focused measurements, such as treatment adherence, internal motivation, or adverse events could be included to help increase understanding of the effects, if any, of SMS from the patients’ point of view. Fifth, an assessment of capacity level needs to be included in a baseline assessment, at least for a random sample of the total. This would provide a means for investigating how capacity might affect results. Sixth, a random sample of the total number of patients could be followed up to help track behavior after the intervention. For this subsample, bill accounting, follow-up calls, responses to 2-way text messages, or nurse or physician assessment of patient participation in treatment would be needed. We suggest this on a random sample, as we feel that such detailed data acquisition could threaten validity by causing attrition. Seventh, to engage vulnerable patients or those living with mental health issues, collaboration with user groups or patient association should be more active, beyond just the consultations or meetings we conducted in this study.

### Conclusions

Undisclosed effects could potentially be further explored in future studies. Perhaps the small suggestion that those in the SMS group were less unwell at readmission is one such upstream effect, rather than just a chance finding. However, in terms of the outcomes that we believe policymakers, clinicians, and patients and caregivers would find important, our tailored, acceptable SMS technique was ineffectual.

## Appendix

Multimedia Appendix 1 CONSORT EHEALTH checklist V1.6.1.

## Abbreviations

CSQ-8: Client Satisfaction Questionnaire-8
GAS: Global Assessment Scale
HILMO: Care Register for Health Care
ICD-10: International Classification of Diseases, 10th Revision
ICT: information and communication technology
OR: odds ratio
Q-LES-Q: Quality of Life Enjoyment and Satisfaction Questionnaire
RR: relative risk
SMS: short message service
